# Spatio-temporal analyses of cropland degradation in the irrigated lowlands of Uzbekistan using remote-sensing and logistic regression modeling

**DOI:** 10.1007/s10661-012-2904-6

**Published:** 2012-10-03

**Authors:** Olena Dubovyk, Gunter Menz, Christopher Conrad, Elena Kan, Miriam Machwitz, Asia Khamzina

**Affiliations:** 1 Center for Development Research, University of Bonn, Walter-Flex Str. 3, 53113 Bonn, Germany; 2 Remote Sensing Research Group, Department of Geography, University of Bonn, Meckenheimer Allee, 166, 53115 Bonn, Germany; 3 Remote Sensing Unit, Institute of Geography, University of Würzburg, Am Hubland, 97074 Würzburg, Germany; 4 ZEF/UNESCO Project, Urgench State University, Khamid Alimjan str., 14, 220100 Urgench, Uzbekistan

**Keywords:** Cropland abandonment, Linear trend analysis, Logistic regression modeling, MODIS, NDVI, Lower reaches of Amu Darya River

## Abstract

Advancing land degradation in the irrigated areas of Central Asia hinders sustainable development of this predominantly agricultural region. To support decisions on mitigating cropland degradation, this study combines linear trend analysis and spatial logistic regression modeling to expose a land degradation trend in the Khorezm region, Uzbekistan, and to analyze the causes. Time series of the 250-m MODIS NDVI, summed over the growing seasons of 2000–2010, were used to derive areas with an apparent negative vegetation trend; this was interpreted as an indicator of land degradation. About one third (161,000 ha) of the region’s area experienced negative trends of different magnitude. The vegetation decline was particularly evident on the low-fertility lands bordering on the natural sandy desert, suggesting that these areas should be prioritized in mitigation planning. The results of logistic modeling indicate that the spatial pattern of the observed trend is mainly associated with the level of the groundwater table (odds = 330 %), land-use intensity (odds = 103 %), low soil quality (odds = 49 %), slope (odds = 29 %), and salinity of the groundwater (odds = 26 %). Areas, threatened by land degradation, were mapped by fitting the estimated model parameters to available data. The elaborated approach, combining remote-sensing and GIS, can form the basis for developing a common tool for monitoring land degradation trends in irrigated croplands of Central Asia.

## Introduction

In the Central Asian countries of the Aral Sea Basin (ASB), about 22 million people depend on irrigated agriculture, while the downstream countries Uzbekistan and Turkmenistan use about 80 % of the water from the ASB (2008). In the predominantly arid climate in the ASB, the expansion of the agricultural areas from about 4.5 Mha in 1960 to almost 7.9 Mha by 1999 was made possible through the construction of massive irrigation systems (Saiko and Zonn 2000). The land appropriation of irrigated agriculture, mostly for cultivation of the water-consuming cash crop cotton, took its toll on the natural land and water resources in the region. The result is the Aral Sea shrinkage due to irrigation water withdrawal from the tributary rivers, rising groundwater tables and subsequent soil salinization (Spoor and Krutov 2003). The land degradation (LD) due to salinity has plagued about 75 % of the irrigated area of the ASB (van Dijk et al. 1999), leading to reduced productivity of the arable land and, eventually, its withdrawal from agriculture. The annual losses in Uzbekistan due to LD were estimated at US$ 31 million, while withdrawal of highly salinized land out of agricultural production cost US$ 12 million (World Bank 2002). The international concern about LD in Central Asia led to the launch of the Subregional Action Programme for the Central Asian Countries on Combating Desertification (UNCCD 2003). Despite the recognized severity of the problem, information on the long-term changes in the state of the land has yet to be collected to develop a common assessment and monitoring system in the region (Dregne 2002). The demarcation of the irrigated cropland areas affected by LD, and identification of direct and proximate causes of LD would make it possible to target areas for mitigation efforts and to prioritize those in need of immediate policy attention.

Among various techniques to detect LD trends, remote-sensing provides a cost-effective evaluation over large areas, whereas in-situ process studies are resource demanding and thus usually conducted at a small scale (Gao and Liu 2010). Mapping of LD can be achieved by studying spatio-temporal dynamics of land-use and land-cover (LULC) changes (e.g., Kessler and Stroosnijder 2006; Lu et al. 2007; Zhang et al. 2008; Biro et al. 2010). This approach implies, among others, image classification (Gao and Liu 2008), spectral mixture modeling (Tromp and Epema 1998), and principal component analysis (Chikhaoui et al. 2005), followed by spatial comparison of the derived maps to quantify changes in degradation classes (Li et al. 2007). For example, Collado et al. (2002) applied spectral mixture modeling to bi-temporal Landsat images to delineate areas affected by desertification in Argentina. Chen and Rao (2008) utilized 3-year Landsat data to map grassland degradation and soil salinization in China, using decision tree classifier and field investigation. In a recent study, Yiran et al. (2011) analyzed LD processes in Ghana by integrating local knowledge with multi-temporal data sets from the LANDSAT Thematic Mapper (TM). Wälder et al. (2008) used geostatistical methods, complemented with expert knowledge for the ecological system modeling and soil mapping in floodplains. Most of these studies focused on natural and semi-natural dryland environments (e.g., Röder et al. 2008). Much less attention was paid to the remote-sensing monitoring of LD in agricultural areas (e.g., Tottrup and Rasmussen 2004), where LD trends can be masked by land management practices, such as irrigation and fertilizer application.

The above-mentioned LULC analyses are suitable for describing spatial changes in land-use classes, but they are not capable of quantifying gradual degradation processes within one land-use class (Röder et al. 2008). Such processes can be captured by trend analyses of multi-year satellite images (Lambin and Linderman 2006; Udelhoven 2011). Trend analyses were routinely employed for LD assessment, using coarse-scale imagery (e.g., Wessels et al. 2007). Only a limited number of studies used satellite time series of medium and high spatial resolution, which are likely to be more appropriate for monitoring of fragmented landscapes of drylands (Sonnenschein et al. 2011). The reason is that the medium-scale data, for example from the AQUA/TERRA-Moderate Resolution Imaging Spectroradiometer (MODIS), until recently did not cover sufficiently long periods to allow trend analyses (Fensholt and Proud 2012; Prince et al. 2009). The comparatively higher-scale images from the LANDSAT program, recorded since 1972, are not always in place for all geographical areas, e.g., Central Asia, on the frequent and repeatable basis required for trend analyses. The current availability of over-decade MODIS imagery gives an opportunity to advance LD monitoring of irrigated drylands.

Despite a considerable amount of literature available on the subject of LD, only a few studies have explicitly linked this phenomenon with its factors (Gao and Liu 2010). Some studies implemented statistical analyses to correlate observed trends with individual drivers. For example, Bai and Dent (2009) analyzed relationships between degraded areas and LULC, population density, aridity index, and poverty in China. Vlek et al. (2008) correlated LD in sub-Saharan Africa with population, terrain, soil, and LULC. The relative importance of factors contributing to the spread of LD in irrigated agricultural regions has been less studied. Information on the relevant LD factors can be gathered by integration of remote-sensing techniques and spatial statistical modeling. In irrigated agroecosystems such as in the ASB, incorporating farmers’ knowledge could bring additional insights to the LD factors.

In this context, the study had two objectives. First, the LD trend in the irrigated croplands was mapped with the MODIS time series with the example of the Khorezm region of Uzbekistan. Second, logistic regression modeling was used to (1) explain the spatial distribution of degraded areas by analyzing their possible factors, and (2) assess the relative importance of the factors regarding observed degradation. The identified factors were employed to map areas at risk of LD as a means to draw attention to the degraded croplands in urgent need of rehabilitation.

## Study region and data sources

### Study region

**Fig. 1:**
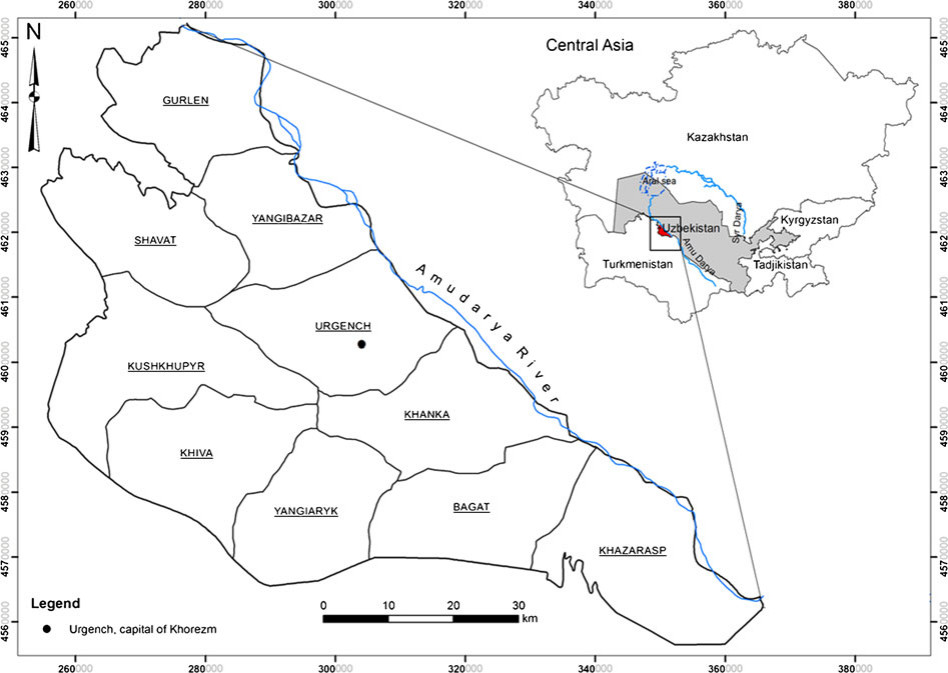
Location of study area in the Khorezm region, Uzbekistan. The region’s border coincides with the extent of irrigated land

The study region of Khorezm is located in the north-western part of Uzbekistan, in the lower reaches of the Amu Darya River. The region consists of ten districts that cover a total area of 560,000 ha with a population of about 1.5 million people (Fig. 1). About 70 % of the population is engaged in crop production and in animal husbandry and horticulture. The region borders on the natural sandy deserts Karakum and Kyzylkum in the south and east, and belongs to the Central Asian semi-desert zone with an extreme, continental climate. The annual precipitation, averaging 100 mm (Tischbein et al. 2012), falls mostly outside the crop-growing season (April–October) and is greatly exceeded by annual evaporation (Conrad et al. 2012). Thus, crop production entirely depends on irrigation.

The irrigated cropland extends over 270,000 ha with an average field size of 2.59 ha. The same crop often grows on the adjacent fields, exceeding the area of 250 ha (Conrad et al. 2011). Most of the arable land is occupied by cotton (60–70 %) and winter wheat (20–30 %) (Shi et al. 2007), cultivated under the state procurement system, where production goals are set for these strategic crops. Cotton has always been produced in Uzbekistan as a means of gaining export earnings, whereas wheat was introduced in the 1990s for national wheat self-sufficiency. Cotton can be rotated with winter wheat, followed by summer crops (Khalikov and Tillaev 2006). On the area of land that is not assigned to cotton, and following the winter wheat, farmers grow maize, sorghum, watermelons, melons, and vegetables (Conrad et al. 2007).

The irrigation water is supplied from the Amu Darya River via a dense network of 16,000 km of irrigation canals. Seasonal salt leaching is practiced for coping with soil salinization and is supported by the 8,000 km network of drainage collectors. Irrigation water supply to crop fields is determined according to the standard guidelines originating in the 1960s (Rakhimbaev et al. 1992). Gravity irrigation prevails in the flat topography of the region, where slopes do not exceed 10 %. Water pumping from irrigation canals is practiced in the elevated areas on the south-western border of the region (Martius et al. 2012). Given its downstream location along the Amu Darya River, Khorezm is one of the final recipients of the river’s water supply and is susceptible to droughts that, during the years 2000, 2001 and 2008, resulted in major crop failures.

Virtually, all the cropland soils in the region are subject to various degrees of salinity, primarily as a consequence of the salt transport from the shallow saline groundwater table, which ranges from 1–1.2 m below the soil surface during salt leaching and irrigation events. Besides poor natural drainage conditions (low-lying location, relief flatness), the shallow water tables result from losses from the irrigation network; the situation is exacerbated by the sub-optimal performance of the drainage system (Ibrakhimov et al. 2007).

### Data sources

The data used in the study include (1) raster data: MODIS images (MOD13Q1, https://lpdaac.usgs.gov/) and (2) vector data: LULC maps for 2001–2009 derived from 250 m MODIS data, infrastructure and environmental data, and (3) ancillary data: datasets of groundwater table and salinity, measured in April, July and October and monthly averaged from 1,798 wells for the period between 1990 and 2004. All raster and vector data were converted to the same coordinate system (ED 1950 UTM Zone 41N). The vector and ancillary data were collected from the ZEF/UNESCO project database (http://www.khorezm.zef.de/), the LULC maps were developed by Machwitz et al. (2010). Interviews were held with the farmers from Khorezm to obtain local perceptions about the LD problem and its drivers in the study region.

## Methods

The analyses were performed in two stages: (1) LD mapping based on the MODIS normalized difference vegetation index (NDVI) time series, and (2) spatial logistic regression modeling. Both stages involved data preparation, i.e., pre-processing of the MODIS images and making a set of factor maps as inputs to the model. The modeling stage included spatial logistic regression analysis comprising of a multicollinearity check, modeling per se, and validation. Subsequently, the model’s results were used to produce a risk map of LD.

Semi-structured interviews were held with 119 farmers from seven districts of Khorezm in September 2009. For the survey, a combination of purposive and snowball sampling methods was applied (Kumar 1999), i.e., initially randomly sampled respondents were asked to nominate another farmer in the neighborhood who had been farming for at least several years, possibly on degraded land.

### Linear trend analysis

Trend analysis of remote-sensing time series has been used to effectively describe a vegetation trend in natural environments (Sonnenschein et al. 2011) and agricultural ecosystems similar to the presented case (Tottrup and Rasmussen 2004; Fuller 1998). Degradation in drylands manifests itself in the reduced productive potential of the land (Reynolds et al. 2007). In arid and semi-arid areas, the sum of the NDVI over a growing season (∑NDVI) is strongly correlated with the vegetation production (Nicholson et al. 1998), revealing that a decreasing linear trend is a good indicator of the vegetation loss for an early warning of LD (Wessels et al. 2004; Budde et al. 2004).

The time series of NDVI images, acquired from the MODIS MOD13Q1 product (collection 5) at a resolution of 250 m × 250 m for the period 2000–2010, were used. The MODIS data were selected due to their more effective spatial and temporal resolution in comparison with other remote-sensing imagery and their spanning over a longer period of time (Section 1). The MOD13Q1 datasets are atmospherically corrected (Vermote et al. 2002) and composed of the best observations during 16-day periods with regard to overall pixel quality (aerosol content, low view angle, and absence of clouds/cloud shadows) and observational coverage (Justice et al. 2002).

**Fig. 2:**
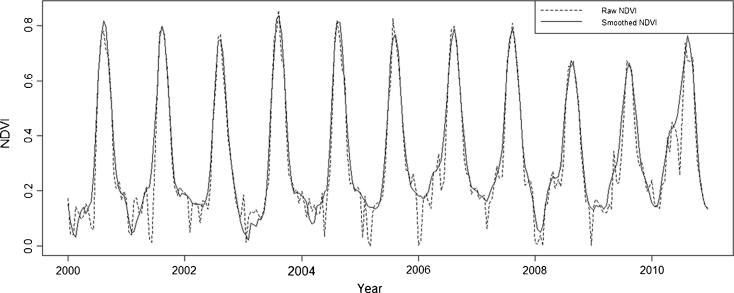
Raw and smoothed 16-day, 250 m MODIS NDVI time series of one pixel

The data were pre-processed by (1) identifying and removing low-quality pixels based on the data quality flags specified in MOD13Q1, (2) filling data gaps with linear interpolation, and (3) smoothing images with an adaptive Savitsky–Golay filter (Fig. 2; Jönsson and Eklundh 2002). During the smoothing procedure, the data quality flags were applied to weigh the data; higher weights were assigned to higher-quality pixels, while lower-quality data had a minor influence on the curve fit. The advantage of the data pre-processing is visible in Fig. 2 when comparing the original NDVI time series with the filtered data.

The smoothed NDVI images were summed over the crop-growing season (April–October) for every year between 2000 and 2010 and served as an input for the linear regression analysis. The analysis focused on the irrigated lands (Fig. 1). All other areas, e.g., water bodies, settlements, were masked out.

For each pixel on the map, slope and intercept of the linear regression were calculated. The intercept characterizes the level of vegetation cover estimated at the date of the first image in the time series. The slope shows the direction and magnitude of the vegetation changes over the analyzed period of time. As these parameters are calculated pixel-wise, the derived temporal changes can be shown in a spatially differentiated way (Röder et al. 2008).

The statistical robustness of the estimated trend was tested with a *T* test. The class boundaries were defined for 90 and 95 % confidence levels. The resulting trend map was regrouped into four classes (Table 1). Analysis of neutral and positive slopes of the linear trend was outside the scope of this study, where the main research focus was on LD.

**Table 1:** Definition of classes for mapping the negative vegetation trend in the Khorezm region of Uzbekistan

Class name	Class boundary
High negative vegetation trend	*T* values of the negative slope > 0.025*
Medium negative vegetation trend	*T* values of the negative slope > 0.05*
Low negative vegetation trend	*T* values of the negative slope < 0.05*
Other	Other slope values

*p values of both tails of the distribution

The quality of the calculated trends can be evaluated by assessing the impact of the errors associated with a particular image and its position in the time series (Hostert et al. 2003). The effect is greater at the beginning and end of the time series, while errors in the middle of the series have less influence on the direction of the trend (Röder et al. 2008). To test an impact on the trend by individual scenes, the calculated trend map was compared to the full set of 11 ∑NDVI images and 2 reduced sets of 10 images without the 2000 and 2011 scenes. In addition, direct field observations were conducted for validation of the derived trend map. Altogether, 186 fields, representing four classes (Table 1), were randomly sampled in summer 2011.

### Spatial logistic regression modeling

#### Data compilation for logistic regression

The list of factors determining LD in the study area was summarized based on interviews with local experts and a review of literature for the Khorezm region (e.g., Ibrakhimov et al. 2007; Akramkhanov et al. 2011). The identified factors ranged from soil, groundwater, and relief characteristics to land ownership and management. The main factors for which the data were available served as inputs to the logistic regression model (Table 2).

**Table 2:** Variables included in the spatial logistic regression model

Variable		Description	Nature of variable
I Dependent	*y*	Degraded land (1—degraded land, 0—not)	Binary
II Independent:			
(a) Site-specific characteristics			
Change in land use	*x*_1_	Change in land use (1—no change, 0—change)	Binary
Uncultivated land	*x*_2_	Uncultivated land (1—lack of cultivation; 0 –cultivation)	Binary
Soil *bonitation* I	*x*_3_	Class I “very high” (1—class I, 0—other classes)	Binary
Soil *bonitation* II	*x*_4_	Class II “increased” (1—class II, 0—other classes)	Binary
Soil *bonitation* III	*x*_5_	Class III “average” (1—class III, 0—other classes)	Binary
Soil *bonitation* IV	*x*_6_	Class IV “low” (1—class IV, 0—other classes)	Binary
Canal density	*x*_7_	Density of irrigation canals (m/m^2^)	Continuous
Collector density	*x*_8_	Density of drainage collectors (m/m^2^)	Continuous
Water use	*x*_9_	Average delta of water use per district (million m^3^)	Continuous
Slope	*x*_10_	Slope (%)	Continuous
Groundwater table	*x*_11_	Level of groundwater table (m)	Continuous
Groundwater salinity	*x*_12_	Groundwater salinity (g/l)	Continuous
(b) Proximity characteristics			
Distance to canals	*x*_13_	Distance to irrigation canals (m)	Continuous
Distance to collectors	*x*_14_	Distance to drainage collectors (m)	Continuous
Distance to pumps	*x*_15_	Distance to water pumps (m)	Continuous
Distance to roads	*x*_16_	Distance to roads (m)	Continuous
Distance to settlements	*x*_17_	Distance to settlements (m)	Continuous
Distance to water bodies	*x*_18_	Distance to lakes and the Amu Darya River (m)	Continuous

The corresponding factor maps were prepared for each factor (independent variables *x*_*i*_). The nature of the maps was binary (presence of a factor = 1, absence = 0) and continuous; they had the same spatial extent, 250 m × 250 m cell size, map projection, and coordinate system. The binary map for the dependent variable (*y*) was represented by the significant negative ΣNDVI trend through merging the classes “high negative vegetation trend” and “medium negative vegetation trend” into a new class “degraded land” (Section 3.1).

The site-specific characteristics included land use (change in land use, lack of cultivation), soil suitability for crop production (as determined by the local scale soil *bonitation*), density of irrigation and drainage network, irrigation water use, slope, and groundwater table level and salinity. The information on land-use change and lack of cultivation were derived from the LULC maps for 2001–2009 based on post-classification comparison. The areas were described as “no change” areas when the agricultural land use remained the same for at least 6 years. The map of uncultivated croplands was similarly derived, indicating areas that were abandoned from cropping for 6 years during the observation period.

Soil *bonitation* is a quantitative soil fertility indicator, introduced in the Soviet Union (Karmanov 1980) and still relevant in a number of ASB states, to assess the land suitability for cropping, using cotton as the reference crop in the assessment (Ramazonov and Yusupbekov 2003). It is an aggregate of several parameters, including field characteristics and soil-inherent properties, e.g., soil texture, organic matter content. Values range from 0 to 100 points with values <40 classifying low-fertility soils (Table 2).

The maps of groundwater table level and salinity were derived via spherical kriging interpolation based on values averaged over the years 1990–2004 for 1,798 observation points as suggested by Ibrakhimov et al. (2007). These authors showed that groundwater table and groundwater salinity did not significantly fluctuate over the years except for the drought year 2000. Thus, the 1990–2004 data were assumed a reasonable approximation for the time period 2000–2010 covered by the NDVI analysis.

Available shapefiles of irrigation and drainage network were used to calculate the density of canals and drains. Factor maps depicting distances to roads, settlements, irrigation canals, drainage collectors, and water bodies were derived based on the Euclidean distances. The water use, showing differences in water supply, was calculated per district for each pair of years between 2000 and 2010 and averaged over the eleven years.

#### Logistic regression

Coupled with GIS, logistic regression is an appropriate tool for explanatory analysis of the factors of LULC changes (Menz et al. 2010). We applied this model to quantify the contribution of the LD factors and to identify areas at risk of LD. Spatial distribution of LD was explained as a function of these factors (Table 2). The nature of LD was regarded as binary, where values 1 and 0 were used to denote its presence and absence, respectively. Consequently, the probability of LD occurring was computed with a logistic regression model (Eq. 1) (Hosmer and Lemeshow 2000):

1 $$ P(y) = 1/1 + \exp { -^{{\left( {\beta_0 + \sum\nolimits_{i = 1}^n {\beta_i {x_i}} } \right)}}} $$

where *P*(*y*) is probability of the dependent variable *y* being 1 given the independent factors *x*_1_…*x*_*n*_, *ß*_0_ is an intercept of the model, *ß*_*i*_ (with 1 < = *i* < = 18) are estimated model parameters, which can be interpreted by analyzing odds of the model (Rothman et al. 2008).

To avoid multicollinearity among model predictors, variance inflation factors (VIF) were calculated, and correlated factors were removed when VIF exceeded the threshold value of 5 (Belsley et al. 1980). The sample size for logistic regression of 8,112 observations resulted from the systematic unbalanced random sampling with a 3 × 3 cell window (750 m × 750 m). Sampling was applied to minimize the impact of spatial dependency between observations, which might cause unreliable estimation of the model parameters (Irwin and Geoghegan 2001). The sample was equally divided into calibration and validation datasets. The former was used to fit the logistic regression, following a backward stepwise procedure. The resulting stepwise model was compared to the ordinary model with a relative operation characteristic (ROC) test (Hanley and McNeil 1982), which checks the equality of the ROC area of each modality. The best-performing model was selected to generate the LD risk map.

#### Model validation

The statistical measures ROC and percentage of correct predictions (PCP) were calculated to evaluate the model performance (Christensen 1997). The ROC value ranges from 0 to 1, where 1 indicates a perfect fit, 0.5 indicates a random fit, while values between 0.5 and 1 show some association between dependent and independent variables (Pontius and Schneider 2001). The PCP is defined as the percentage of correctly predicted pixels to the total number of pixels in the map.

For validation, the final model was applied to the validation dataset, and the probability of LD was computed for every pixel with the fitted logistic regression model (Eq. 1). The ROC and PCP were used for comparison of the actual degradation (Section 3.2) and computed probabilities. In the case of the PCP, the modeled degradation was assigned to the pixels, i.e., if the probability exceeded a commonly accepted threshold value of 0.5, the cell was marked as degraded land (Manel et al. 1999). In addition, the goodness of fit was evaluated with chi-square statistics (Moore and McCabe 1998).

## Results

### Linear trend analysis

**Fig. 3:**
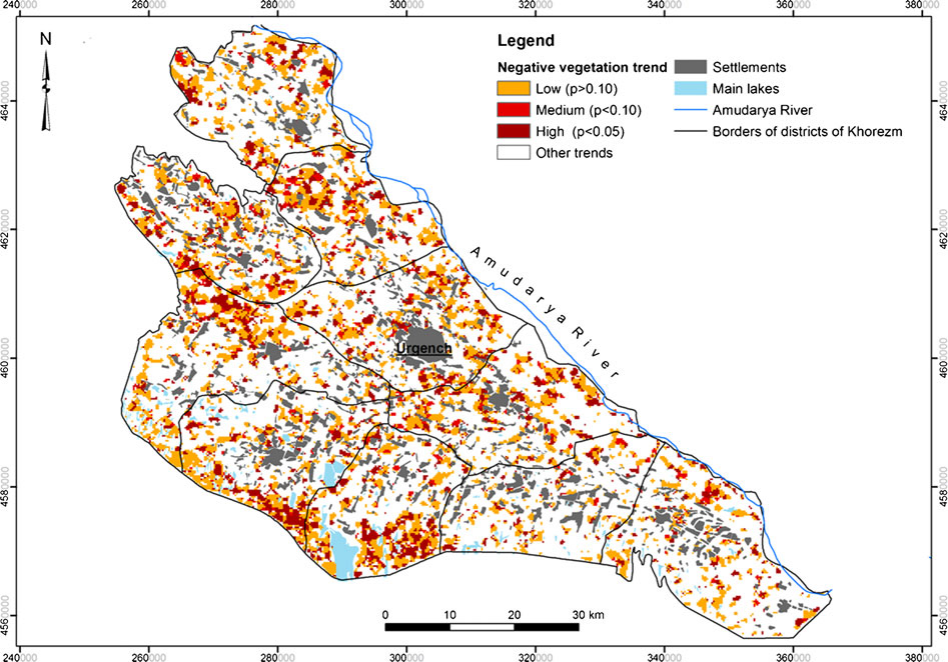
Negative vegetation trend in the Khorezm region of Uzbekistan, calculated from slope of linear trend of NDVI time series, summed over the growing seasons 2000–2010

The resulting ΣNDVI-based trend map highlights areas that experienced constant vegetation losses during the monitoring decade (Fig. 3). For each pixel in the map, the retained value was the slope of the fitted linear regression between the values of each pixel over time and a perfectly linear time series; thus, the results express the rate of vegetation loss per observation year.

A gradual negative vegetation trend of different magnitude was observed. Overall, its spatial distribution was highly variable, but several clusters were distinguished in the southwest (Yangiaryk and Khiva districts) and northwest (north of the Kushkhupyr, Yangibazar, and Shavat districts) of the region. The lands, located mostly on the outskirts of the irrigated cropland area, were characterized by a relatively low vegetation cover at the beginning of the observation period, and experienced gradual vegetation losses thereafter. Most of these areas are dominated by sandy soils, which are less suitable for crop production. Smaller degraded patches were scattered throughout the region and did not show any particular spatial pattern.

The areal statistics of LD, expressed by a negative vegetation trend, are shown in Table 3. About 33 % of the study area experienced degradation processes of low, medium, and high magnitude during 2000–2010. The areas with the low and high negative trend yielded higher percentages compared to the area with the medium-magnitude trend.

**Table 3:** Areal statistics per district of Khorezm for different classes of degradation in percent

District	Low		Medium		High		High and Medium	
	(ha)	(%)	(ha)	(%)	(ha)	(%)	(ha)	(%)
Bagat	7,398.91	16.67	979,90	2.21	2,865.47	6.46	3,845.37	8.66
Gurlen	9,636.70	22.92	1,110.32	2.64	3,529.58	8.39	4,639.90	11.04
Khanka	10,394.84	22.89	1,913.06	4.21	5,126.04	11.29	7,039.10	15.50
Khazarasp	9,415.64	18.07	1,147.32	2.20	3,839.87	7.37	4,987.19	9.57
Khiva	10,080.17	21.77	1,429.34	3.09	5,754.96	12.43	7,184.29	15.52
Kushkhupyr	11,981.58	21.91	1,649.20	3.02	5,792.80	10.59	7,442.00	13.61
Shavat	10,040.41	24.07	1,644.60	3.94	5,006.26	12.00	6,650.86	15.94
Urgench	10,897.64	22.74	1,617.68	3.38	5,278.79	11.01	6,896.48	14.39
Yangiaryk	10,102.87	24.59	1,456.93	3.55	6,054.39	14.74	7,511.32	18.28
Yangibazar	8,096.92	22.65	1,726.44	4.83	4,280.70	11.97	6,007.14	16.80
TOTAL	98,044.69	21.72	14,674.78	3.25	47,528.87	10.53	62,203.65	13.78

The cross validation, implemented between two pairs of the trend maps (trend map, based on the full set of images and two reduced sets; Section 3.1), yielded overall accuracies of 87 and 91 %, while omitting images from 2000 and 2010, respectively. This confirms the robustness of the calculated trend. The validation of the trend map, based on the field data, yielded an overall accuracy of 78 %.

### Spatial logistic regression modeling

#### Model statement and interpretation

After the multicollinearity check, the final list of LD factors (Table 2) was reduced by one variable, i.e., soil *bonitation* IV “low” (*x*_6_), with the corresponding VIF value of 6.23. Two models were built: the full model incorporating all variables, and the reduced model resulting from the backward stepwise procedure. The ROC test yielded a significant result with *p* values < 0.05 (*α* = 0.05), suggesting a difference in prediction power between these models. Thus, the full model was employed for logistic regression. The final full model was significant with chi-square values of 801.11 and corresponding *p* values < 0.001 (*α* = 0.05). The model validation results with a ROC value of 0.70 suggested a good prediction power, exceeding a random assignment by 20 %. The PCP of 69 % indicated higher than average agreement between predictions and reality.

The logistic regression ruled out statistically insignificant variables, including all *bonitation* classes (*x*_3_, *x*_4_, and *x*_5_), density of canals and collectors (*x*_7_, *x*_8_), and distance to collectors, pumps, and water bodies (*x*_14_, *x*_15_, and *x*_18_) (Table 4).

**Table 4:** Estimated parameters of logistic regression model

Variable		Coefficient (*ß*_*i*_)	Odds ratio, %	Standard error	*z*	*p* > |*z*|
Change in land use	*x*_1_	0.14	14.76	0.08	2.04	**
Uncultivated land	*x*_2_	0.71	102.72	0.26	5.59	****
Soil *bonitation* I	*x*_3_	0.40	48.59	0.62	0.95	n.s
Soil *bonitation* II	*x*_4_	0.01	1.16	0.08	0.13	n.s
Soil *bonitation* III	*x*_5_	−0.12	11.34	0.07	−1.57	n.s
Soil *bonitation* IV	*x*_6_	Omitted due to multicollinearity				
Canal density	*x*_7_	−0.00	−0.27	0.00	−1.45	n.s
Collector density	*x*_8_	0.00	0.00	0.00	0.41	n.s
Water use	*x*_9_	−0.09	−9.71	0.02	6.86	****
Slope	*x*_10_	0.25	28.96	0.15	2.23	**
Groundwater table	*x*_11_	1.46	329.73	0.65	9.71	****
Groundwater salinity	*x*_12_	0.23	25.96	0.07	4.13	****
Distans to canals	*x*_13_	0.08	8.39	0.02	4.91	****
Distance to collectors	*x*_14_	−0.02	−1.49	0.02	0.62	n.s
Distance to pumps	*x*_15_	0.00	0.28	0.00	0.65	n.s
Distance to roads	*x*_16_	0.04	3.62	0.01	−4.81	****
Distance to settlements	*x*_17_	−0.02	−1.75	0.01	2.06	**
Distance to water bodies	*x*_18_		1.57	0.01	0.63	n.s.
Constant	*ß*_i_	−3.55	–	0.16	−22.33	****

*n*.*s*. not significant

*p < 0.1; **p < 0.05; ***p < 0.01; ****p < 0.001

In accordance with the estimated model parameters, level of groundwater table, land without cultivation, slope, and groundwater salinity had the strongest impact on the spatial distribution of LD in Khorezm (Table 4). Specifically, the degraded areas were associated with the land that was abandoned from cultivation for six or more years, and were characterized by a deeper groundwater table level and steeper slopes. The odds of LD were 329.73, 102.72, and 28.96 % higher on land with the deeper groundwater level, uncultivated land, and areas with steeper slopes, respectively, than on other lands. These results correlate with the observed clusters of negative vegetation trend and are also in line with the farmers’ opinions; the farmers mentioned poor soil quality (sandy soils), lack of water, and steep slopes as the reasons for LD in these areas. There, irrigation water is supplied up to the elevated areas via pumps, which are not in use when maintenance and electricity costs cannot be afforded.

**Fig. 4:**
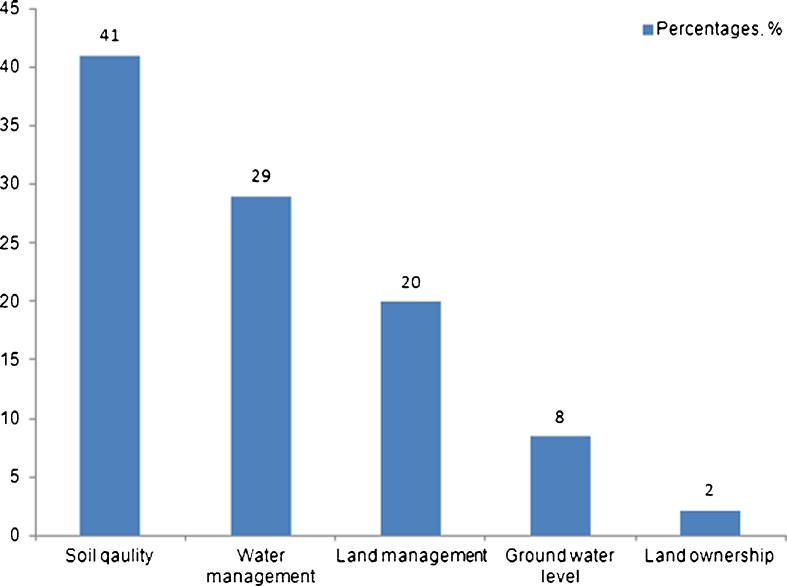
Farmers’ opinion on factors of land degradation in the Khorezm region of Uzbekistan. The percentages indicate the frequency of the farmers’ replies

The importance of groundwater salinity was reflected by the odds of the factor *x*_12_, suggesting that an increase in groundwater salinity by 1 g/l increases the chance of LD by 25.96 %. The availability and distribution of water were also observed to influence the spatial patterns of LD. The negative relation with the factor water use (*x*_9_) showed that degraded areas tended to occur in the districts with shorter water supplies. The areas further away from the irrigation canals (factor *x*_13_) were more prone to degradation. LD dependence on the vicinity to roads (*x*_16_) indicated that easily accessible lands were better managed (Fig. 4). Though the estimated odds of the factor distance to settlements (*x*_17_) showed a negative relation to degradation, its small value indicated a comparatively weak influence on the observed spatial patterns.

#### Factors of land degradation as perceived by farmers

All surveyed farmers acknowledged the problem of cropland degradation on their farms. From the land-user perspective, soil quality was the main factor of LD (Fig. 4), including sandy texture and, in 28 % of all replies, soil salinity. Most of the respondents stated that soils with low *bonitation* were particularly prone to degradation. Inadequacies of water management (lack of timely supplies of irrigation water, poor maintenance of the irrigation system and water pumps), and land management (absence of crop rotation, lack of land reclamation measures) were next in importance. A few farmers indicated a high groundwater table and groundwater salinity as factors. In 2 % of the cases, the lack of land ownership was considered an indirect trigger of LD.

#### Mapping areas at risk of land degradation

**Fig. 5:**
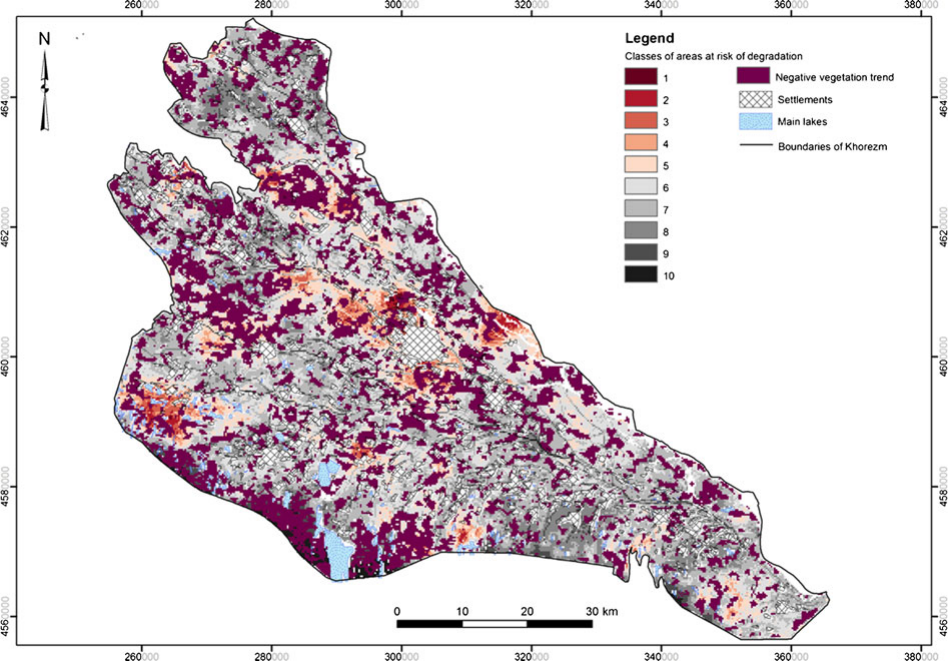
Risk map of land degradation in the Khorezm region of Uzbekistan. Class 1 indicates areas with the highest risk of degradation that gradually reduces to class 10. *Dark violet* areas represent land with negative vegetation trend, derived from trend analysis of 250 m MODIS NDVI time series

Spatial patterns of land at risk of LD were derived by applying the estimated coefficients of the model to the factor maps following Eq. 1. The resulting map was reclassified into ten classes, allocating sequentially 10 % of total probability values per class (i.e., 10 % of the highest probability values are grouped in class 1) (Fig. 5).

Several clusters of areas at risk of LD (classes 1 to 5) were predicted: central part of the region near the capital, north of the region (border between the Gurlen and Yangibazar districts), Kushkhupyr district, and the southern parts of Khorezm bordering the Karakum Desert. The rest of the region was classified as have a medium to very low risk of LD (classes 6 to 10).

## Discussion

The natural settings in Khorezm, including arid climate, flat terrain with enclosed saline lakes and depressions, soils poor in major nutrients, and hydromorphic soils, are favorable to LD, particularly so under sub-optimal land and water management practices (Martius et al. 2012). The results of the spatio-temporal analysis confirm the occurrence of irrigated cropland degradation, which stemmed from a combination of environmental and anthropogenic factors.

### Mapping of land degradation trend

In this study, the LD trend was analyzed based on a decrease in the vegetation cover which, in land-use systems, may also occur due to changes in land management (Bai and Dent 2009). In Uzbekistan, the land-use decisions largely remained unchanged during the study period given the area-based, state production targets for cotton and the prevalence of cotton and winter wheat in the cropland area (Djanibekov et al. 2010; Shi et al. 2007). Furthermore, summing the NDVI over the whole growing season, thus integrating vegetation peaks in the fields with different land uses, reduced the possibility of misinterpretations, particularly for the remaining land fraction with a variable cropping pattern. This approach was previously applied in studies conducted in arid and semi-arid cropland environments (Fuller 1998; Tottrup and Rasmussen 2004).

In contrast to the cropping pattern, the effect of irrigation management on NDVI, and thus LD trend, cannot be assumed constant. Although the standard guidelines are reportedly followed for crop irrigation, the regional water supplies fluctuate from year to year, drastically decreasing during seasonal and long-term droughts (Tischbein et al. 2012). Nevertheless, the existing yield quotas assigned for the dominant crops allow assuming that, to fulfill these production targets in drought years, the fertile croplands are prioritized in leaching and irrigation decisions rather than the areas of low *bonitation*. Such strategy is likely to aggravate the LD processes occurring in these areas.

A very close association (*R* ^2^ = 0.98) of LD hotspots and the soil *bonitation* class IV, that characterizes soils with inherently low fertility, confirmed the low suitability of these lands for cropping. As revealed by the calculated trend on desert margins in the southwest, these low-*bonitation* croplands experienced the strongest decline in vegetation cover and were abandoned. Recent studies in the region show that the alternative use of these areas for afforestation with native tree species could increase the productive and economic potential of the land (Djanibekov et al. 2012; Khamzina et al. 2012).

The results of the accuracy assessment confirm the validity of the elaborated approach, suggesting its applicability to regional LD monitoring. The robustness of the calculated LD trend was comparable with that observed in other dryland studies using trend analysis (e.g., Hostert et al. 2003; Röder et al. 2008). The accuracy of the trend map, based on direct field observations, was similar or higher than the accuracies reported in related studies. For example, Chen and Rao (2010) yielded an overall accuracy of 65 % for the regional LD map derived from the MODIS data in a transition zone between grassland and cropland in northeast China.

The use of time series with a finer spatial resolution than the 250 m MODIS data could disclose an additional level of information, particularly considering the patchy structure of the agricultural landscape in Khorezm. With respect to the direction of the LD trend and its landscape patterns, results from coarse and fine resolution imagery are expected to correspond, based on the experience of Stellmes et al. (2010) in Mediterranean drylands.

### Factors of land degradation

In explaining the LD trend with logistic regression, the influence of contiguous areas with the relatively deep groundwater tables outweighed that in scattered land patches with a shallower groundwater table. This contrasts with the expected impact of a shallow groundwater table, which causes soil salinization and thus LD. However, given that the deeper groundwater tables were observed on croplands, abandoned from cultivation for at least 6 years, a deepened groundwater table can be a consequence of reduced irrigation inputs. The groundwater levels, observed in these locations, remained above the critical threshold of 2 m (Ibrakhimov et al. 2007), thus still posing a risk of soil salinization and, therefore, decline in crop growth. Akramkhanov and Vlek (2011) in Khiva district of Khorezm also identified higher soil salinity when the groundwater table was deeper, and attributed this phenomenon to great differences in capillary fluxes in various soil textures.

Generally, very few studies have analyzed the impact of environmental and management factors on LD trends in irrigated croplands. Akramkhanov et al. (2011) focused on the spatial distribution of soil salinity at the farm scale in Khiva district of Khorezm. The study, confined to the year 2002, revealed a low, though a significant, correlation with band 7 of LANDSAT TM, distance to drainage collectors, and the groundwater parameters, thus suggesting that management practices, particularly water management, outweighed the impact of environmental factors on the pattern of soil salinity. In a following study, Akramkhanov and Vlek (2011) used an artificial neural network as an alternative to the regression technique, and detected that soil salinity distribution was influenced by the micro-topographical features, which tended to affect surface water retention. The observed correlations with the remote-sensing parameters and groundwater depth and salinity (Akramkhanov et al. 2011) are in line with the results of the presented regional assessment. The contrasts can be explained by the different spatial as well as by the different temporal scales of the analyses, given that crop production decline and LD due to salinity only becomes obvious in the long run, as annual leaching practices counterbalance the salinization process.

The importance of water management for the distribution of soil salinity at farm scale (Akramkhanov and Vlek 2011) was also indicated by the results of the farmer survey, thus confirming the significance of this factor at the local level. Most of the surveyed farmers attributed declined yields not only to soil salinity but also to other soil and terrain properties. Gray and Morant (2003) suggested caution in interpreting farmers’ opinions in environmental assessments due to observed discrepancies with formal, scientific data on soil quality. Sulieman (2008), who had analyzed causes of agricultural land degradation and abandonment in Sudan, indicated that the value of local knowledge depends on its accuracy, which cannot be fully verified without independent sources of information but, is of significant value in case of no or limited availability of the scientific data. A future study should investigate how and why differences in information emerge and reconcile them for a more efficient use of the local knowledge in assessment of LD trends.

The prediction power of the elaborated model, reflected in PCP and ROC values, is comparable to the previously reported studies for ecological and LULC applications of logistic regression. For example, Manel et al. (1999) reported PCP values in the range of 67–81 %, and Pontius and Schneider (2001) reported ROC values of 65–70 %. The present results from the model highlight its main advantages such as spatial explicitness and quantitative analysis of the factors. Moreover, predictions are possible based on the observed relationships, as also mentioned by Koomen and Stillwell (2007). The model’s prediction results were conditioned to the incorporated variables, which were assumed to represent the most important factors influencing the spatial distribution of LD. Incorporation of more variables was subject to data constraints, a common issue for LULC models (Dubovyk et al. 2011). Aiming to provide a regional overview, the derived risk map renders a visual representation of areas under risk that could be prioritized in more detailed analyses and the attention of decision makers.

## Conclusions

The MODIS data were found suitable for regional-scale monitoring of negative vegetation trends, which can be interpreted in relation to LD. The results of the linear trend analysis of the MODIS NDVI time series reveal a degradation trend in the study area during 2000–2010.

The LD hotspots were predominantly found in the outskirts of the irrigation system, in the margins bordering natural deserts, and in areas locally classified as least suitable for cropping. The degradation processes tend to exacerbate the situation due to lack of cultivation. Abandoned croplands should therefore be the main target of rehabilitation measures or considered for alternative uses.

The applied integrated approach, combining spatial logistic regression and trend analysis of satellite time series, allowed the inclusive evaluation of irrigated cropland degradation at the regional scale. The model made it possible to explain the factors of the observed trend and to map areas at risk of LD that could be targeted in a finer resolution assessment. The elaborated approach can be further developed for monitoring of LD trends in irrigated croplands of Central Asia.
